# First-in-man ZIPPER™ endograft system for the treatment of symptomatic aortic arch intramural haematoma

**DOI:** 10.1093/ehjcr/ytad574

**Published:** 2023-11-17

**Authors:** Honglin Dong, Weiguo Fu, Wayne W Zhang

**Affiliations:** Department of Vascular Surgery, 2nd Affiliated Hospital of Shanxi Medical University, Taiyuan, Shanxi, China; Department of Vascular Surgery, Zhongshan Hospital, Fudan University, Shanghai, China; Division of Vascular and Endovascular Surgery, Department of Surgery, University of Washington School of Medicine, 1959 NE Pacific Street, Box 356410, Seattle, WA 98195, USA

A 55-year-old woman presented with sudden onset of chest tightness and upper back pain. Computed tomography angiography (CTA) revealed intramural haematoma (IMH) involving the aortic arch and extending into the abdominal aorta (*Panels A* and *B*).

**Figure:**
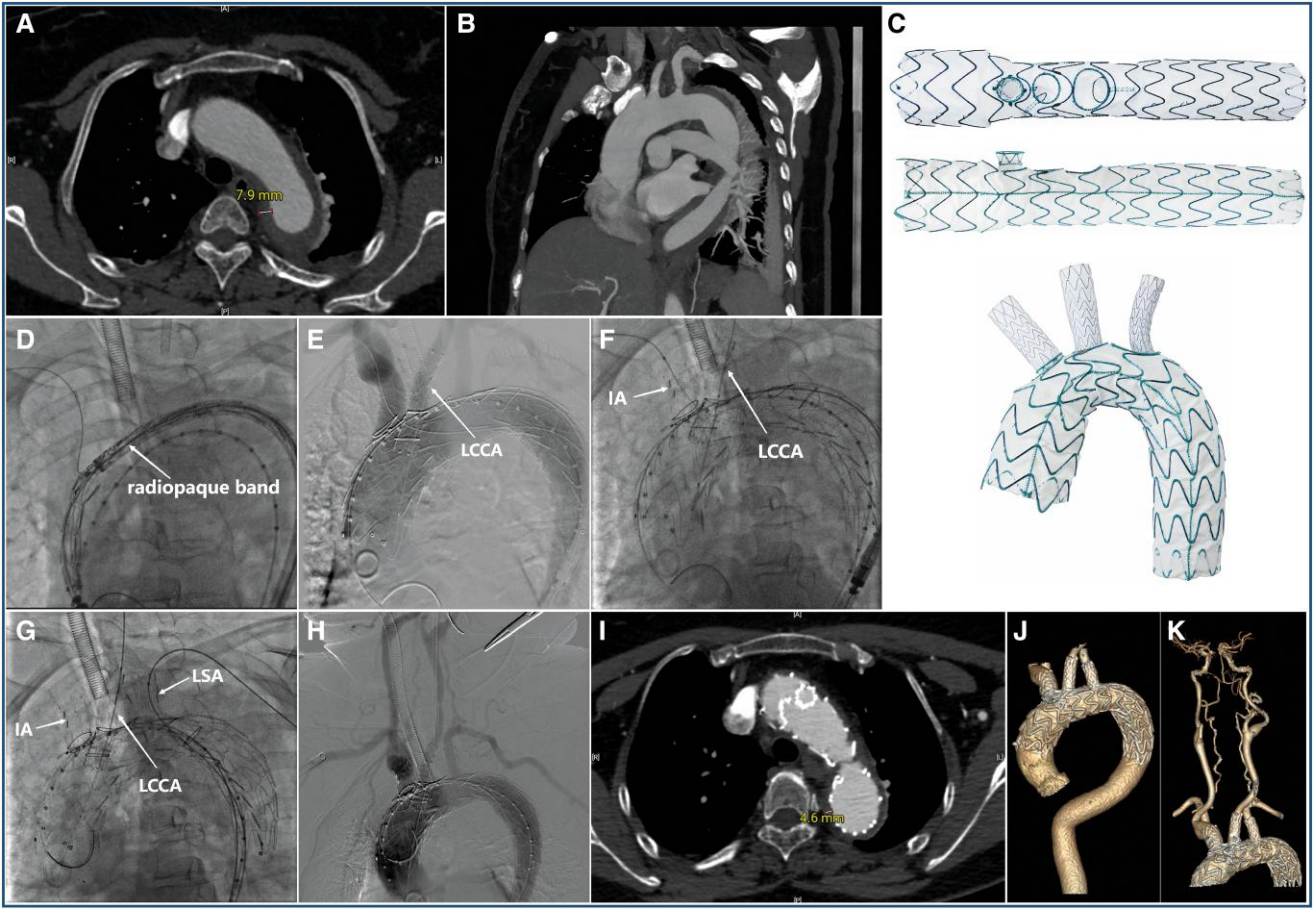


Currently, there is no consensus regarding the best treatment choice of persistent symptomatic aortic arch IMH.^1–3^ Because of persistent upper back pain following 3-week conservative management, treatment strategies including open surgery vs. endovascular repair were discussed with the patient and family. They decided to proceed with endovascular procedure, and a written consent was obtained.

This novel ZIPPER™ endograft system (Hangzhou Endonom Medtech Co., Ltd, China) consists of a main-body stent graft, one outer/inner convertible innominate artery (IA) branch stent graft, and two inner branches for the left common carotid artery (LCCA) and the left subclavian artery (LSA; *Panel C*).

Key procedure steps were as follows: a 22-F steerable delivery system was inserted through the right femoral artery over a 0.035 inch super stiff wire into the aortic arch. A pre-loaded 0.025 inch guidewire via IA branch was snared and pulled through the right brachial access (see Supplementary material online, *Video S1*). The main-body stent graft (38 × 28 × 220 mm) was placed to ensure the second branch aligned with LCCA (*Panel D*). The LCCA was retrogradely reconstructed with a stent graft (10 × 11 × 50 mm; *Panel E*; Supplementary material online, *Video S2*), followed by IA stenting (16 × 14 × 40 mm) via the right brachial artery (*Panel F*; Supplementary material online, *Video S3*). The LSA was then stented with a covered stent (10 × 11 × 50 mm) through the femoral artery approach (*Panel G*; Supplementary material online, *Video S4*). Angiography confirmed that the main-body and branch stent grafts were widely patent (*Panel H*).

The patient was discharged home 1 week after surgery with resolved symptoms. Computed tomography angiography at 1 month showed IMH regression (*Panel I*) and no stent graft–related complications (*Panel J* and *K*).

## Supplementary Material

ytad574_Supplementary_Data

## Data Availability

The data underlying this article are available within the article and in its online Supplementary material. Additional data will be shared upon reasonable request to the corresponding author.

## Notes

**Consent:** The authors confirm that written consent for submission and publication of this case report including images and associated text has been obtained from the patient in line with COPE guidance.

**Funding:** None declared.

## References

[1] Brown JA , Arnaoutakis GJ, Kilic A, Gleason TG, Aranda-Michel E, Sultan I. Current trends in the management of acute type A aortic intramural hematoma. J Card Surg 2020;35:2331–2337. 32652687 10.1111/jocs.14819

[2] Spanos K , Kölbel T. Treatment of intramural haematoma of the ascending aorta (type A) should be selective. Eur J Vasc Endovasc Surg 2021;62:7–8. 34024712 10.1016/j.ejvs.2021.04.012

[3] Vacirca A , Dias Neto M, Baghbani-Oskouei A, Huang Y, Tenorio ER, Estrera A, et al Timing of intervention for aortic intramural hematoma. Ann Vasc Surg 2023;94:14–21. 36309166 10.1016/j.avsg.2022.09.041

